# The role of gestational diabetes, pre-pregnancy body mass index and gestational weight gain on the risk of newborn macrosomia: results from a prospective multicentre study

**DOI:** 10.1186/1471-2393-14-23

**Published:** 2014-01-15

**Authors:** Salvatore Alberico, Marcella Montico, Valentina Barresi, Lorenzo Monasta, Caterina Businelli, Valentina Soini, Anna Erenbourg, Luca Ronfani, Gianpaolo Maso

**Affiliations:** 1 Department of Obstetrics and Gynaecology, Institute for Maternal and Child Health - IRCCS “Burlo Garofolo”, via dell’Istria 65/1, 34137 Trieste Italy; 2 Epidemiology and Biostatistics Unit, Institute for Maternal and Child Health - IRCCS “Burlo Garofolo”, via dell’Istria 65/1, 34137 Trieste Italy

**Keywords:** Gestational diabetes mellitus, Pregnancy, Body mass index, Weight gain, Newborn macrosomia

## Abstract

**Background:**

It is crucial to identify in large population samples the most important determinants of excessive fetal growth. The aim of the study was to evaluate the independent role of pre-pregnancy body mass index (BMI), gestational weight gain and gestational diabetes on the risk of macrosomia.

**Methods:**

A prospective study collected data on mode of delivery and maternal/neonatal outcomes in eleven Hospitals in Italy. Multiple pregnancies and preterm deliveries were excluded. The sample included 14109 women with complete records. Associations between exposure variables and newborn macrosomia were analyzed using Pearson’s chi squared test. Multiple logistic regression models were built to assess the independent association between potential predictors and macrosomia.

**Results:**

Maternal obesity (adjusted OR 1.7, 95% CI 1.4-2.2), excessive gestational weight gain (adjusted OR 1.9, 95% CI 1.6-2.2) and diabetes (adjusted OR 2.1, 95% CI 1.5-3.0 for gestational; adjusted OR 3.0, 95% CI 1.2-7.6 for pre-gestational) resulted to be independent predictors of macrosomia, when adjusted for other recognized risk factors. Since no significant interaction was found between pre-gestational BMI and gestational weight gain, excessive weight gain should be considered an independent risk factor for macrosomia. In the sub-group of women affected by gestational or pre-gestational diabetes, pre-gestational BMI was not significantly associated to macrosomia, while excessive pregnancy weight gain, maternal height and gestational age at delivery were significantly associated. In this sub-population, pregnancy weight gain less than recommended was not significantly associated to a reduction in macrosomia.

**Conclusions:**

Our findings indicate that maternal obesity, gestational weight gain excess and diabetes should be considered as independent risk factors for newborn macrosomia. To adequately evaluate the clinical evolution of pregnancy all three variables need to be carefully assessed and monitored.

## Background

The incidence of obesity and gestational diabetes (GDM) is rising worldwide. The prevalence of obesity, defined as a Body Mass Index (BMI) equal or over 30 Kg/m2, increased from 12% in 1991 to 17.9% in 1998, in the U.S.A. [1]. Similarly, the overall prevalence of diagnosed gestational diabetes in the U.S. was less than 6% in 1994, while in 2000 approximately half of the States had a prevalence equal to 6%. By 2009, only two American States maintained a similar prevalence while in fifteen States it exceeded 9% [2].

It is already well known that both obesity and GDM are relative risk factors for adverse maternal and neonatal outcomes, being related to an increased occurrence of Large for Gestational Age (LGA) fetuses and macrosomia (defined as neonates birthweight over 4000 g). Although macrosomia can be influenced by both genetic and environmental factors, the rapid increase in prevalence is mainly attributable to environmental causes [3]. Among these, maternal overweight and the related metabolic changes such as diabetes mellitus type 2 and GDM, seem to be crucially important.

A large number of studies have demonstrated a significant association between pre-pregnancy BMI and neonatal birthweight [4]. More recent cohort studies have assessed the effect of gestational weight gain on neonatal birthweight. The majority of these studies were limited by small sample sizes or unreliable data collection methods such as the use of non-validated questionnaires [5,6]. It is recognized that pre-pregnancy BMI and gestational weight gain depend on different metabolic pathways. Pre-pregnancy BMI represents maternal nutritional conditions before conception, while gestational weight gain is the expression of fetal-maternal physiological changes combined with genetic and nutritional factors. The fetal contribution (fetal weight, placenta and amniotic fluid) accounts for 30-40% of maternal weight gain [7], while maternal factors (plasmatic volume expansion, breast and uterine pregnancy growth, maternal fat deposits available for breastfeeding and extracellular fluid volume) account for 60-70% [8].

Evidences suggest that maternal diabetes increases the occurrence of large for gestational age (defined as neonatal birthweight superior to the 90th percentile) and its contribution may lead to incidence rates as high as 28.5%, as demonstrated by the DIEP Study in 1991 [9]. Furthermore, macrosomic neonates born to diabetic and overweight mothers are exposed to a much higher risk of developing young age obesity: 17.1% versus 14.2% in the control group at birth, and 9.7% versus 6.6% at adolescence [10]. A study carried out on a large cohort of pregnant women suggested that suboptimal glycemic control could be an important risk factor for the development of macrosomia, independently of pre-pregnancy BMI [11].

In this context it is crucial to understand which, between pre-pregnancy BMI and pregnancy weight gain, is the most important determinant of excessive fetal growth. Thus, the aim of the present study was to identify in a large sample of women the most important risk factors determining macrosomia and to evaluate the independent role of pre-pregnancy BMI, gestational weight gain and gestational diabetes in increasing the risk of excessive fetal growth.

## Methods

An eighteen-months prospective study collected data on mode of delivery and maternal/neonatal outcomes from eleven single-institutional obstetric cohorts in Friuli Venezia Giulia, a region of North-Eastern Italy accounting for roughly 10,000 deliveries per year. The study was conducted in compliance with the Helsinki Declaration and was approved by the institutional review board of the coordinating center (Technical Scientific Committee of the Institute for Maternal and Child Health - IRCCS “Burlo Garofolo”, Trieste, project 86/05, February 28, 2007). All women provided written informed consent for the inclusion of their records in the presentation of summary data. Access to the data was approved by all hospital trust administrations. Study methods are described in detail elsewhere [12]. Briefly, the source institutions were first level departments serving low risk pregnancies, except for two centers which have a Neonatal Intensive Care Unit (NICU, second referral units). We created an ad hoc regional computerized database considering maternal characteristics, variables related to pregnancy and mode of delivery. We collected information on maternal characteristics such as maternal age, pre-pregnancy BMI, weight gain during pregnancy and presence of one or more medical or obstetrical conditions including pre-gestational and gestational diabetes. Data on institutional deliveries were prospectively collected at the time of delivery. To maintain overall data completeness and accuracy, during the study period periodical meetings were organized to discuss the results and provide assistance.

All women were screened for gestational diabetes in each centre. In three centers, the screening test was carried out using a two steps procedure: a 50 g Glucose Challenge Test at first and if positive (values higher than 140 mg), patients were offered a 75 g Oral Glucose Tolerance Test. Gestational diabetes was diagnosed if two out of three glycemic values exceeded 95 mg, 180 mg and 155 mg/dl. In eight centers, gestational diabetes was screened through a 100 g Oral Glucose Tolerance Test and diagnosed when any two out of four glycemic values exceeded 95 mg-180 mg-155 mg-140 mg/dl.

Once gestational diabetes was diagnosed, patients were recommended to carry out a glycemic profile collecting fasting glucose and 1-hour postprandial values. Insulin treatment was offered if fasting glucose values exceed 90 mg/dl or postprandial values were superior to 120 mg/dl in at least 20% of the evaluations carried out within a week.

The Body Mass Index was calculated as weight in kg/(height in meters)^2^. Data on height, pre-gestational weight and weight at delivery were collected for each woman*.* Pre-pregnancy BMI was classified using the World Health Organization criteria as underweight (inferior to 18.5), normal (18.5 to 24.9), overweight (25.0 to 29.9) and obese (equal or superior to 30). Pregnancy weight gain was classified as defined by Institute of Medicine (IOM) guidelines [13]. According to this statement, recommended pregnancy weight gain was differentiated based on pre-pregnancy BMI: 12.5-18.0 kg for underweight women, 11.5-16.0 kg for normal-weight, 7.0-11.5 kg for overweight and 4.5.0-9.0 kg for obese [13]. The main study outcome was macrosomia, defined as a birthweight equal or above 4000 g, as in previous studies [3-7].

Only cases with a complete collection of the variables described above were included in the statistical analysis. Multiple pregnancies were excluded as women were more likely to gain excess weight and neonatal birthweights were more likely to be lower than in single pregnancies. Preterm deliveries were excluded because women were more likely to gain less weight than in term pregnancies.

Continuous variables were reported as means and standard deviations, while categorical data as absolute frequencies and percentages, odds ratios (OR) and relative 95% confidence intervals (CI). Associations between exposures and outcome variables were analyzed using Pearson’s chi squared test. Spearman’s correlation was used to study the relation of maternal weigh gain and pre-pregnancy BMI with neonatal birthweight. Two multiple logistic regression models were built to assess the independent association of potential predictor variables of macrosomia both in the whole population and in a subgroup of women affected by gestational diabetes. Predictor variables were: maternal age at conception, maternal diseases (autoimmune and hypertensive disorders), pre-gestational BMI, gestational weight gain, parity, maternal diabetes, maternal height, gestational age at delivery, newborn sex. A step-down approach retaining only significant variables was used. A p-value inferior to 0.05 was considered as statistically significant.

## Results

The sample included 14109 women with complete records. Mean maternal age at conception was 31.7 years (SD 5.2). Primiparae accounted for 53.7% of deliveries. At the time of conception 14.5% of women were overweight and 5% obese; 29.1% of women gained more weight than recommended by IOM [13] (Table 1). Gestational age at delivery was beyond 40 weeks in 18.6% of cases. Gestational diabetes was diagnosed in 360 women (2.6%). The proportion of macrosomia was 7.6%. Mode of delivery was cesarean section in 21.4% of cases (Table 1).

**Table 1:** Description of the sample (14109 pregnant women)

**Variable**	**Mean or frequency**
**Maternal age at conception in years, mean (SD)**	31.7 (5.2)
**Categories of maternal age at conception, number (%)**	
<20 years	170 (1.2)
20-35 years	10559 (74.8)
36-45 years	3370 (23.9)
>45 years	10 (0.1)
**Parity, number (%)**	
Primiparous	7577 (53.7)
Multiparous	6532 (46.3)
**Maternal height at conception in cm, mean (SD)**	165.5 (6.1)
**Categories of maternal height at conception, number (%)**	
≤ 165 cm	7461 (52.9)
>165 cm	6648 (47.1)
**Maternal weight at conception in kg, mean (SD)**	62.1 (11.2)
**Maternal BMI at conception, mean (SD)**	22.6 (3.8)
**Categories of maternal BMI at conception, number (%)**	
<18.5	1033 (7.3)
18.5-24.9	10323 (73.2)
25-29.9	2040 (14.5)
≥30	713 (5.0)
**Gestational age at delivery in weeks, mean (SD)**	39 (1.2)
**Categories of gestational age at delivery, number (%)**	
37-40 weeks	11481 (81.4)
>40 weeks	2628 (18.6)
**Maternal diabetes, number (%)**	
No	13713 (97.1)
Pre-gestational	36 (0.3)
Gestational	360 (2.6)
**Maternal weight gain during pregnancy in kg, mean (SD)**	12.9 (4.3)
**Categories of maternal weight gain during pregnancy according to IOM recommendations, number (%)**	
Recommended by IOM	5755 (40.8)
< Recommended by IOM	4248 (30.1)
> Recommended by IOM	4106 (29.1)
**Mode of delivery, number (%)**	
Spontaneous	9974 (70.7)
Operative	1119 (7.9)
Cesarean section	3016 (21.4)
**Infant birthweight in g, mean (SD)**	3377 (430)
**Categories of infant birthweight, number (%)**	
<4000 g	13043 (92.4)
≥4000 g	1066 (7.6)
**Neonates sex, number (%)**	
Female	7006 (49.7)
Male	7103 (50.3)

Data extracted from this sample showed that pre-gestational BMI was significantly related to macrosomia (Table 2). The proportion of macrosomia was 10.4% in overweight women and 15.7% in obese women compared to 6.9% in normal weight women (Chi square for trend p < 0.001). Similarly, 12.6% of the offspring of women with excessive weight gain were macrosomic compared to 6.5% of the offspring of normal weight gain women (p < 0.001). Among women diagnosed with gestational diabetes 14.2% gave birth to macrosomic babies compared to 16.7% among mothers affected by pre-gestational diabetes and 7.4% among healthy women (p = 0.032 and p < 0.001 respectively). Other risk factors significantly related to macrosomia were: parity (9.6% macrosomic babies among multiparous versus 5.8% among primiparous; p < 0.001), maternal height (9.7% among women taller than 165 cm versus 5.6% among women shorter than 165 cm; p < 0.001) and gestational age at delivery (16.9% if beyond 40 weeks versus 5.4% if less than 40 weeks, p < 0.001). Maternal age at delivery was not significantly associated with macrosomia.

**Table 2:** Crude and adjusted odds ratios of developing offspring macrosomia

	**Macrosomia (birthweight >4000 g)**				
	**(%)**	**Crude OR**	**p-value**	**Adjusted OR**	**p-value**
		**(95% CI)**		**(95% CI)**	
Pre-gestational BMI*					
18.5-24.9	(6.9)	1		1	
<18.5	(2.8)	0.4 (0.3 - 0.6)	<0.001	0.5 (0.3-0.7)	<0.001
25-29.9	(10.4)	1.6 (1.3 - 1.8)	<0.001	1.1 (0.9-1.3)	0.319
≥30	(15.7)	2.5 (2.0 - 3.1)	<0.001	1.7 (1.4-2.2)	<0.001
**Gestational weight gain**					
Recommended by IOM	(6.5)	1		1	
< Recommended by IOM	(4.1)	0.6 (0.5 - 0.7)	<0.001	0.6 (0.5-0.8)	<0.001
> Recommended by IOM	(12.6)	2.1 (1.8 - 2.4)	<0.001	1.9 (1.6-2.2)	<0.001
**Parity**					
Primiparous	(5.8)	1		1	
Multiparous	(9.6)	1.7 (1.5 - 1.9)	<0.001	2.0 (1.7-2.3)	<0.001
**Maternal Diabetes**					
No	(7.4)	1		1	
Gestational diabetes	(14.2)	2.1 (1.5 - 2.8)	<0.001	2.1 (1.5-3.0)	<0.001
Pre-gestational diabetes	(16.7)	2.5 (1.0 - 6.1)	0.032	3.0 (1.2-7.6)	0.023
**Maternal height (cm)**					
≤165	(5.6)	1		1	
>165	(9.7)	1.8 (1.6 - 2.1)	<0.001	1. 9 (1.6-2.2)	<0.001
**Gestational age (weeks)**					
37-40	(5.4)	1		1	
>40	(16.9)	3.5 (3.1 - 4.0)	<0.001	3. 7 (3.2-4.2)	<0.001
**Neonate sex**					
Female	(5.0)	1		1	
Male	(10.1)	2.1 (1.9 - 2.4)	<0.001	2.2 (2.0-2.6)	<0.001

*Test for Trend: p <0.001.

Low weight gain was significantly related to low birthweight of the offspring (OR 2.0 for low weight gain versus recommended weight gain; 95% CI: 1.6 - 2.8; p < 0.001). Only two women with gestational diabetes gave birth to low birthweight babies.

Correlation of maternal weigh gain and pre-pregnancy BMI with neonatal birthweight resulted irrelevant (Spearman’s correlation rho 0.17 and 0.15, respectively).

A multivariate logistic regression was carried out to evaluate the independent association between exposure variables and the outcome macrosomia. Diabetes confirmed its role as independent predictor of macrosomia, with a 2.1-fold increased risk of macrosomia among women with gestational diabetes compared to women without diabetes, and of 3.0-fold in women with pre-gestational diabetes (Table 2).

Obesity was found to be an important independent predictor of macrosomia: obese women had a 1.7-fold increased risk of developing offspring macrosomia compared to normal weight women (Adjusted OR 1.7; 95% CI 1.4-2.2; p < 0.001). Maternal overweight was not significantly associated to macrosomia, once adjusted for the other risk factors (Adjusted OR 1.1; 95% CI 0.9-1.3; p = 0.319).

Once adjusted for other risk factors, the odds of developing macrosomia among women whose weight gain in pregnancy was more than recommended by IOM compared to women whose gain was in the recommended range was 1.9 (95% CI 1.6-2.2; p < 0.001).

Maternal height above 165 cm, gestational age at delivery greater than 40 weeks and male sex of the neonate were independent predictors of macrosomia (Table 2). No significant interaction between pre-pregnancy BMI and gestational weight gain was found.

A subgroup analysis was carried out on women affected by gestational diabetes (Table 3). Among these patients, pre-gestational BMI was no longer significantly associated to macrosomia. An excessive pregnancy weight gain was related to a 2.6-fold increased risk of developing macrosomia when compared to recommended weight gain (adjusted OR 2.6; 95% CI 1.2-5.5; p = 0.018). Maternal height greater than 165 cm (adjusted OR 3.3; 95% CI 1.7-6.4; p < 0.001) and gestational age at delivery greater than 40 weeks (adjusted OR 4.5; 95% CI 2.0-10.1) were independent predictors of newborn macrosomia. Maternal gestational weight gain lower than recommended was not significantly associated with a reduction in macrosomia (adjusted OR 0.8; 95% CI 0.3-1.8; p = 0.541).

**Table 3:** Subgroup analysis on women with gestational diabetes: crude and adjusted odds ratios of developing offspring macrosomia

	**Macrosomia (birthweight ≥ 4000 g)**			
	**Crude OR**	**p-value**	Adjusted OR*	**p-value**
	**(95% CI)**		**(95% CI)**	
**Gestational weight gain**				
Recommended by IOM	1		1	
> Recommended by IOM	2.3 (1.1 - 4.8)	0.017	2.6 (1.2-5.5)	0.018
< Recommended by IOM	0.9 (0.4 - 2.0)	0.777	0.8 (0.3-1.8)	0.541
**Maternal height (cm)**				
≤165	1		1	
>165	2.9 (1.5 - 5.4)	<0.001	3.3 (1.7-6.4)	<0.001
**Gestational age (weeks)**				
37-40	1		1	
>40	3.6 (1.7 - 7.6)	<0.001	4.5 (2.0-10.1)	<0.001

*Adjusted for pre-gestational BMI, parity, sex of the offspring.

## Discussion

Our findings suggest that maternal obesity, excessive gestational weight gain and diabetes are independent valuable predictors of macrosomia, when adjusted for other recognized risk factors (parity, mother’s height, gestational age at birth, neonate sex). No significant interaction was found between pre-gestational BMI and gestational weight gain. Among women affected by gestational or pre-gestational diabetes, pre-gestational BMI was no longer significantly associated to macrosomia, while excessive pregnancy weight gain, maternal height and gestational age at delivery were independent risk factors to predict macrosomia. In this population, pregnancy weight gain lower than recommended was not significantly associated with a reduction in macrosomia.

Pre-gestational BMI is a universally recognized factor affecting fetal growth [14]. In our study, obese women are more likely to develop offspring macrosomia, with 1.7-fold risk increase compared to normal weight women, thereby confirming the results of previous published studies [14,15]. Contrary to what has been previously suggested [16], our overweight women did not have an increased risk of developing macrosomia: once results were adjusted for gestational weight gain and other recognized risk factors for excessive fetal growth, the odds of developing macrosomia was 1.1 (95% CI 0.9-1.3; p = 0.319) compared to normal weight patients. A possible explanation for these results could be that among overweight patients, gestational weight gain is a more crucial factor in increasing the risk of macrosomia. In fact, in a recent study by Di Benedetto A et al., the incidence of macrosomia among overweight women with a normal gestational weight gain was around 4.8%, which is similar to that in normal weight women with a normal gestational weight gain, while in overweight patients with excessive weight gain the incidence of macrosomia was up to 13% [16]. In any case, no continuous relation was found between pre-gestational BMI and neonatal birthweight.

Excessive gestational weight gain has been extensively proven to be an important risk factor for the development of macrosomia, independently of pre-gestational BMI [17,18]. This finding is confirmed by our results, that support the crucial role of pregnancy weight gain, according to IOM cut-offs, in determining fetal weight in the general population, regardless of nutritional and diabetic status. In our population of pregnant women, gestational weight gain below the recommended range was found to be protective with respect to macrosomia (adjusted OR 0.6; 95% CI 0.5-0.8; p < 0.001), but was also associated with an increased incidence of low birthweight babies (crude OR 2.0 for low weight gain versus recommended weight gain; 95% CI 1.6-2.8). The observed association confirmed previously published results, offering strong evidence to support the association between excessive gestational weight gain and increased fetal growth and, at the other end of the scale, decreased weight gain and the development of small for gestational age babies [4,19]. Since we did not find a continuous relation between maternal weight gain and neonatal weight, the cut-offs defined by IOM seem to be appropriate in classifying maternal weight gain.

Since we did not find any significant interaction between pre-pregnancy BMI and gestational weight gain, excessive weight gain should be considered as an independent risk factor for macrosomia for both non-obese and obese women. These findings are confirmed by another recent study [17]. Neither of these studies classifies obese women by severity of disease and this could perhaps explain the lack of interaction. This represents a limitation of our study, because the severity of obesity has been proven to modify the association between gestational weight gain and fetal growth [20]. In other terms, the more severe the obesity of a patient the lower tends to be the weight gained during pregnancy.

Subgroup analysis among women with gestational diabetes showed that excessive gestational weight gain played an independent role in increasing the risk of macrosomia in this group (adjusted OR 2.6; 95% CI 1.2-5.5). Furthermore in this subgroup of patients, a lower than recommended gestational weight gain did not significantly reduce the risk of developing macrosomia (OR 0.8; 95% CI 0.3-1.8). These results are in contradiction with previous findings by other authors, suggesting that a further reduction in the weight gain recommended by IOM could decrease the incidence of macrosomia and guarantee optimal neonatal outcome [21,22]. Indeed, some authors have suggested that IOM recommendations do not respond to the needs of obese women affected by gestational diabetes and should only be applied to obese, non-diabetic pregnant patients [22]. In our subgroup population, pre-pregnancy overweight and obesity were unrelated to macrosomia, but this result needs to be verified with a larger population.

A very careful re-assessment of ideal gestational weight gain among obese women affected by gestational diabetes should be carried out, as also suggested by literature. Although among obese patients, a gestational weight gain below IOM recommendations was associated with decreased odds of large for gestational age offspring, it was also significantly related to increased odds of small for gestational age neonates [23]. Furthermore, in this population, women who gained weight according to IOM guidelines were not exposed to an increased risk of delivering a low birthweight infant [24]. Among obese and overweight women affected by gestational diabetes, a third trimester weight loss was found to be associated with decreased odds of macrosomia, but increased odds of small for gestational age offspring [25]. The most important limitation of our study consists in the lack of adjustment for glycemic control, since data on glycemia were not available. In fact, the study was originally developed to explore the relationship between mode of delivery and maternal/neonatal outcomes. For the same reason, data were not collected on pregnant women using insulin. Despite this limitation, we believe our findings offer an important contribution on this matter. In our population of women with GDM, pre-pregnancy overweight and obesity are unrelated to macrosomia and excessive gestational weight gain plays a stronger role in determining this neonatal outcome. Furthermore, a gestational weight gain less than recommended does not significantly reduce the risk of developing macrosomia, thereby suggesting that hypocaloric diets should be avoided, given the related risk of ketonemic episodes [26] and the consequent possible negative effects on the infant’s intelligence [27].

## Conclusions

Our findings indicate that maternal obesity, excessive gestational weight gain and diabetes should be considered independent risk factors for newborn macrosomia. Therefore, to adequately evaluate the clinical evolution of pregnancy, all three variables need to be carefully assessed and monitored. In the subgroup of women affected by gestational diabetes, excessive gestational weight gain was the main variable related to macrosomia and lower than recommended weight gain did not significantly reduce the risk of developing macrosomia.

## Abbreviations

BMI: Body mass index; CI: Confidence interval; GDM: Gestational diabetes; IOM: Institute of medicine; LGA: Large for gestational age; NICU: Neonatal intensive care unit; OR: Odds ratio.

## Competing interests

The authors declare that they have no competing interests.

## Authors' contributions

SA conceived and designed the study, contributed to research conduction and data collection, interpreted the data, wrote and reviewed the manuscript; MM analyzed and interpreted the data, wrote and reviewed the manuscript; VB contributed to research conduction and data collection; LM analyzed and interpreted the data, reviewed the manuscript; CB contributed to research conduction and data collection; VS contributed to research conduction and data collection; AE interpreted the data, wrote, reviewed and edited the manuscript; LR contributed to research conduction and to data analysis, interpreted the data, wrote and reviewed the manuscript; GM conceived and designed the study, contributed to research conduction and data collection, interpreted the data, wrote and reviewed the manuscript. The collaborators grouped under the name of “Multicentre Study Group on Mode of Delivery in Friuli Venezia Giulia” contributed to the conduction of the research and to data collection. All authors contributed to the drafting of the paper and approved the final version.

## Pre-publication history

The pre-publication history for this paper can be accessed here:

http://www.biomedcentral.com/1471-2393/14/23/prepub
